# Reprogramming Tumor-Associated Macrophage Using Nanocarriers: New Perspectives to Halt Cancer Progression

**DOI:** 10.3390/pharmaceutics16050636

**Published:** 2024-05-09

**Authors:** Alyona B. Kuznetsova, Ekaterina P. Kolesova, Alessandro Parodi, Andrey A. Zamyatnin, Vera S. Egorova

**Affiliations:** 1 Scientific Center for Translation Medicine, Sirius University of Science and Technology, 354340 Sochi, Russia; alyona.kuznetsova.2002@mail.ru (A.B.K.); kolesova.ep@talantiuspeh.ru (E.P.K.); parodi.a@talantiuspeh.ru (A.P.);; 2 Faculty of Bioengineering and Bioinformatics, Lomonosov Moscow State University, 119234 Moscow, Russia; 3 Belozersky Institute of Physico-Chemical Biology, Lomonosov Moscow State University, 119992 Moscow, Russia; 4 Department of Biological Chemistry, Sechenov First Moscow State Medical University, 119991 Moscow, Russia

**Keywords:** tumor-associated macrophage, M2 macrophage, macrophage repolarization, nanotherapy, chemotherapy resistance

## Abstract

Cancer remains a significant challenge for public healthcare systems worldwide. Within the realm of cancer treatment, considerable attention is focused on understanding the tumor microenvironment (TME)—the complex network of non-cancerous elements surrounding the tumor. Among the cells in TME, tumor-associated macrophages (TAMs) play a central role, traditionally categorized as pro-inflammatory M1 macrophages or anti-inflammatory M2 macrophages. Within the TME, M2-like TAMs can create a protective environment conducive to tumor growth and progression. These TAMs secrete a range of factors and molecules that facilitate tumor angiogenesis, increased vascular permeability, chemoresistance, and metastasis. In response to this challenge, efforts are underway to develop adjuvant therapy options aimed at reprogramming TAMs from the M2 to the anti-tumor M1 phenotype. Such reprogramming holds promise for suppressing tumor growth, alleviating chemoresistance, and impeding metastasis. Nanotechnology has enabled the development of nanoformulations that may soon offer healthcare providers the tools to achieve targeted drug delivery, controlled drug release within the TME for TAM reprogramming and reduce drug-related adverse events. In this review, we have synthesized the latest data on TAM polarization in response to TME factors, highlighted the pathological effects of TAMs, and provided insights into existing nanotechnologies aimed at TAM reprogramming and depletion.

## 1. Introduction

Macrophages are essential immune cells that can be found in various tissues throughout the body. They play a crucial role in our immune defense by performing functions such as the direct clearance of invading pathogens through phagocytosis, presenting antigens to activate specific immune responses, and producing cytokines to regulate the immune response. Moreover, these cells participate in wound healing, tissue remodeling and repair [1]. Macrophages dispatch their mission in various organs and tissues: they include tissue Kupffer cells, microglia in the brain, alveolar macrophages in the lungs, Langerhans cells in the skin, splenic macrophages, peritoneal macrophages, etc. [2].

Tumor-associated macrophages (TAMs) are defined as a separate population of macrophages, making up the majority of non-cancerous cells in the tumor protective and supportive microenvironment (TME), which also includes fibroblasts, myelogenic suppressor cells, lymphocytes, the extracellular matrix components and surrounding tumor tissues blood vessels [3]. Tumor-associated macrophages are classified as pro-inflammatory, or classically activated M1 macrophages, and anti-inflammatory, or alternatively activated, M2 macrophages, which play in important role in cancer progression, in particular in tumor angiogenesis, chemotherapy, and immunotherapy resistance and metastasis [4]. As the tumor progresses, M2 macrophages become the dominant cell type in the tumor microenvironment, and, consequently, further accelerate the advancement of the disease with their activity. In this context, there is a need to fine-tune the TAM phenotype and re-educate them towards the M1 phenotype. This reprogramming could boost a patient’s immune system and improve the ability to combat cancer.

In this review we will take a close look at the phenotypes of TAMs, their modulation under the effect of TME factors, such as hypoxia and acidosis, and different available therapy options—in particular, selective and targeted therapy options provided by nanomedicine. Nanotechnology has revolutionized the field of cancer therapy by offering innovative tools for targeted drug delivery and controlled drug release, encapsulation of therapeutic agents and therefore, mitigation of systemic toxicity. With the advancement of nanotherapeutics, precise manipulation of the TME components like TAMs has become a plausible strategy. Nanocarriers can be designed to specifically target TAMs, modulating their phenotype from pro-tumorigenic (M2-like) to anti-tumorigenic (M1-like), thus inhibiting cancer progression and metastasis and preventing the development of chemoresistance in cancer cells.

This review provides state-of-the-art knowledge about polarization of TAMs under the effect of TME factors, the pathogenic role of TAMs in cancer progression, and special attention is paid to development of various nanotherapeutic options to reprogram TAMs in more benign phenotype to combat cancer progression.

## 2. Macrophage Phenotype Plasticity

**Figure 1:**
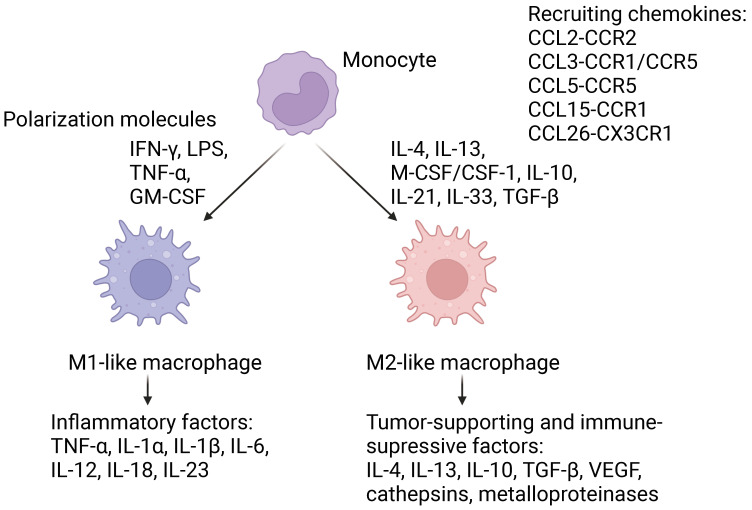
Tumor-associated macrophage recruitment and polarization. Figure was created using BioRender.com, according to [6].

In the human body TAMs are derived mainly from the circulating blood monocytes, which are recruited by signaling molecules, produced by tumor cells and TAMs, which have already undergone activation and polarization [3,5]. These signaling molecules include cytokines, such as IL-4, IL-13, M-CSF/CSF1, IL-10, IL-33, IL-21 and transforming growth factor-β (TGF-β), and chemokines, such as CCL2, CCL3, CCL15, CCL18, CX3CL1, CXCL8, and CXCL12 (Figure 1, for more information see review [6]). Depending on the functions performed by macrophages, they are traditionally divided into two types: classically activated by inflammatory stimuli, such as bacterial liposaccharide (LPS) and IFNγ, M1 macrophages, which participate in Th1-mediated immune response, provide antibacterial protection, and present of antigens using MHCII. To protect tissues against bacteria M1 macrophages induce the inflammation process by producing inflammatory cytokines, such as tumor necrosis factor *α* (TNF-α), IL-1α, IL-1β, IL-6, IL-12, IL-23, producing increased amounts of inducible nitric oxide synthase (iNOS), and, consequently, increasing concentration of NO in extracellular matrix, and producing reactive oxygen species (ROS). The distinctive phenotypic markers of M1 are HLA-DR, CD80, CD86, and MHCII [7]. M1 polarization is also characterized by activation of the transcription factor signal transducer and activator of transcription 1 (STAT1).

Alternatively activated M2 macrophages are polarized by cytokines, such as IL-4, IL-10, IL-13, and transforming growth factor β (TGF-β). These type of cells are also called anti-inflammatory and are responsible for Th2-mediated humoral response against helmints. Another main function of M2 macrophages is tissue healing and reconstruction, for example reconstruction of bone tissue [8]. To realize this function cells overexpress arginase 1, enabling utilization of arginine with the formation of polyamines and proline which can be used during cell proliferation and protein, in particular collagen synthesis [7]. A distinctive feature of M2 is the expression of a number of scavenger receptors, such as CD163, CD204, MARCO (macrophage receptor with collagenous structure) [9], mannose receptor 1 (CD206, MRC1), folate receptor, transferrin receptor, on their cell surface, which also serve as the phenotypic markers of M2 [10,11]. The signaling pathways underlying polarization of M2 macrophages will be discussed in more detail in the next chapter devoted to polarization under the influence of TME factors.

However, it is widely accepted that this binary classification of macrophages on anti-cancer M1 and pro-cancer M2 is oversimplified and can be seen only in vitro as a result of stimulation of activated monocytes with bacterial LPS/IFNγ or IL-4. Single cell RNA sequencing data indicate that TME is enriched by TAMs, expressing both M1 and M2 markers under the effect of tumor producing signaling molecules and different environmental factors in the TME, such as hypoxia and acidosis (effect of hypoxia and acidosis is discussed in Section 2 of this review) [12,13]. At the first stage of the tumor development activity of M1 macrophages, in particular, increased levels of ROS, NO, and the active inflammation process help them to destroy tumor cells, But as the tumor progresses and produces recruiting and polarization factors, such as IL-34, colony stimulating factor 1 (CSF1), M2-like macrophages start to predominate in the TME, which prohibit anticancer response and promote tumor growth and progression, contributing to proliferation and migration of cancer cells, angiogenesis, and metastasis (see Section 3 of this review).

## 3. Factors Affecting Macrophage Polarization in Tumor Microenvironment

### 3.1. Hypoxia

Hypoxia, or oxygen deprivation, is a common characteristic of solid tumors and is closely associated with their malignant progression. Under hypoxic conditions, the level of hypoxia-inducible factor HIF1α increases; it translocates into the nucleus and dimerizes with HIF1β, resulting in activation of HIF1 transcription factor. In turn, HIF1 activates genes responsible for tumor angiogenesis, progression, and metastasis [14,15,16]. One of the primary mechanisms driving tumor progression involves hypoxic conditions, which prompt tumor cells to release extracellular vesicles containing various types of RNAs, including micro, circular RNAs, and long non-coding RNAs. These vesicles, in turn, facilitate the polarization of tumor-supporting M2 macrophages. An illustrative example is the presence of miR-934 in exosomes originating from colorectal cancer cells [17], circ_001381, miR3184-3p, and miR-1246, isolated from cells and cerebrospinal fluid in glioma [18,19,20]. Extracellular vesicles, isolated from glioma cells in hypoxic conditions, contained high levels of IL-6 and miR155-3p, promoting M2 macrophage polarization via the IL-6-pSTAT3-miR-155-3p-autophagy-pSTAT3 pathway and, consequently, glioma progression [21]. Exosomes, derived from esophageal squamous cell carcinoma cells in hypoxic conditions, contained hsa-circ-0048117 [22], which can mediate TLR4 activation, and miR-21-5p, which mediated downregulation of PTEN in cancer cells and co-cultured macrophages, resulting in activation of PI3K/AKT/STAT6 signaling pathway and polarization of macrophages towards M2. The same signaling pathway is activated by miR-21, which is overrepresented in the exosomes of bladder cancer cells [23]. The existence of feedback regulation was also noted: extracellular vesicles, derived from M2 macrophages, also contained miR-21-5p, which mediated the expression of EMT-associated genes: N-cadherin, α-SMA, Snail, and p-SMAD2 in tumor cells [24]. Exosomes isolated from A549 lung cancer cells under hypoxic conditions contain PKM2 protein, one of glycolysis regulators, that mediated activation of AMPK/p38 signaling pathway in TAMs, contributing to M2 polarization [25].

### 3.2. Acidosis

One more distinctive feature of a tumor associated with oxygen deficiency is the occurrence of aerobic glycolysis in tumor cells, resulting in the accumulation of lactate as the end product and, consequently, the acidification of TME (the so-called Warburg effect) [26]. According to estimations, lactate level in TME is about 40 times higher than in normal tissues [27]. However, it is widely known that lactate is not only a glycolysis byproduct in tumor but also plays an important signaling role. Increased acidity of TME is associated with increased tumor progression, aggressiveness, and metastasis rate [28]. One of the mechanisms by which lactate enhances tumor aggressiveness is its ability to reprogram TAMs towards M2 phenotype via the signaling pathway AKT/ERK [26,29,30]. M2 TAMs, in turn, promote the expression of genes responsible for proliferation, survival, and EMT in cancer cells. For example, M2 macrophages polarized under the effect of lactate produced by CRC cells released CCL8, which activated EMT genes in tumor cells via the CCL8/CCR5/70S6K/4EBP1 axis [29]. In breast cancer (BC), M2 macrophages produced CCL17 that induced EMT gene (N-cadherin, vimentin and PCNA) expression in tumors via CCL17/CCR4/mTORC1 axis [31]. The same effect was observed for succinate—one of the major metabolites of cancer cells: succinate induced M2 polarization via PI3K/AKT/HIF1α signaling and promoted EMT and proliferation gene expression in pituary adenocarcinoma cells [32]. Concerning the interaction of hypoxia and acidosis, stabilization of hypoxia factor HIF1α was demonstrated in macrophages under the effect of tumor-derived lactate and succinate in hypoxic and normoxic conditions, which results in production of VEGF and, consequently, enhanced tumor angiogenesis [33,34]. Hepatocellular carcinoma (HCC) and pancreatic cancer cells were shown to produce transcription factor Nuclear Factor (erythroid-derived 2)-Like 2 (Nrf2) under the effect of lactate, which resulted in increased expression of Nrf2 in TAMs, and VEGF production and VEGF stimulated expression of Nrf2 and EMT genes in cancer cells [35]. Novel data disclose another mechanism of lactic acid regulation of macrophage polarization: as a result of the metabolism of lactate in the citric acid cycle, citrate and acetyl-CoA are produced, which contribute to histone H3K27 acetylation. H3K27 acetylation reduces the accessibility of chromatin in pro-inflammatory gene locuses and suppresses the function of their enhancers, resulting in suppression of macrophage inflammatory response [36]. Another mechanism which is supposed to follow histone acetylation in macrophages is histone lactylation, which induces the expression of homeostatic genes that are involved in wound healing, including Arg1 (the M2-like phenotype) [37].

**Figure 2:**
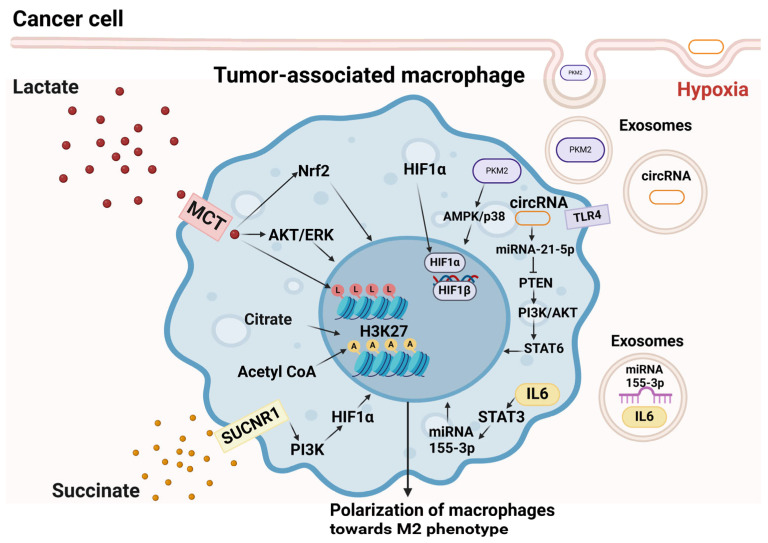
Signaling pathways prompting M2 macrophage polarization under the effect of acidosis and hypoxia in TME. MCT = monocarboxylate transporter 1; SUCNR1 = succinate receptor; see Section 2 for more details. Figure was created with BioRender.com.

Mechanisms of TAM polarization under the effect of hypoxia and acidosis are schematically represented in Figure 2.

## 4. Pathological Effects, Related to Tumor-Associated Macrophage Activity

### 4.1. TAMs Promote Angiogenesis

TAMs are the major producers of different angiogenic factors, in particular, vascular endothelial growth factor (VEGF) [38], placental growth factor (PIGF) [39], and platelet-derived growth factor (PDGF)-BB [40]. VEGF contributes to early stages of angiogenesis and neovascularization, resulting in formation of monolayer leaking endothelial tubes. The later stage of angiogenesis—maturation of primitive vessels—is controlled by activation of angiopoietin and tyrosine kinase Tie-2 system, while Tie-2 is expressed on the surface of the so-called Tie2-expressing macrophages (TEM) [38]. TEMs are represented in tumor regions, in particular, their distribution correlates with neovascularization regions but are absent in non-cancerous regions. It was shown that targeting TEM (for example, using new Tie2 inhibitor rebastinib) in combination with chemo- or immunotherapy is a promising approach to mitigate tumor progression and metastasis [41,42].

Angiogenesis in tumor regions and vascular permeability are closely related to metastasis: formation of vessels and increased permeability of new-formed vessel walls enable migration of cancer cells and formation of distant metastases. The study [43] provides evidence of an interaction between miRNA-23a-3p, downregulation of PTEN, EMT, and metastasis. By targeting PTEN miR-23a-3p increased the expression of VEGF-A, the proliferation and metastasis of HUVEC cells, and angiogenesis. MiR-23a-3p downregulated TJP1, occludin, and claudin 5, leading to increased vascular permeability. The phenomenon of increased vascular permeability in the presence of TAMs was also demonstrated in vitro in 3D system [44]: in the presence of TAMs, in particular M2, endothelium permeability increased 1.5–2 times in comparison with the vessel permeability in the absence of TAMs. Co-culturing of endothelial cells and breast cancer cells with M2 macrophages were associated with increased vascular permeability, resulted from downregulated expression of intercellular junction protein PECAM-1 in the endothelial cells. Enhanced vascular sprouting resulted from increased levels of angiogenic factors, including TGF-β, bFGF, EGF, VEGF-A, IL-8, increased levels of MMP9 in culture medium which was associated with enhanced extracellular matrix porosity, and tumor emboli intravasation into the vessel [44].

Interaction between colorectal cancer cells and TAMs was described in [40]. According to results of the analysis of database data and patient samples, a correlation between increased levels of expression of the transcription factor RUNX1, involved in the control of angiogenesis and hematopoiesis, chemokine CCL2 in CRC cells, and upregulation of M2 expression markers was found. It has been empirically demonstrated that CRC cells mediated TAM recruitment via overproduction of RUNX1, CCL2 and IL-10, and M2 polarization via Hedgehog signaling activation. M2 TAMs, in turn, produce platelet-derived growth factor (PDGF)-BB, which contributes to angiogenesis in tumors and, moreover, enhances production and nuclear translocation of RUNX1 in CRC cells [40].

### 4.2. TAMs Promote Chemotherapy Resistance

Among the mechanisms of cancer chemoresistance development metabolic reprogramming, in particular, autophagy enhancement, activation of cancer stem cells, activation of anti-apoptotic signaling (MAPK, PI3K-AKT, Notch, Wnt signaling) [45,46,47], and production of anti-apoptotic proteins (i.e., Bcl-2) [48,49] are noted. Moreover, overexpression of transporters—which eliminate drugs from the tumor cells, in particular, upregulation of ATP-binding cassette (ABC) transporters—was demonstrated in breast cancer cells [50].

One of the simplest mechanisms that explain participation of M2 TAMs in chemoresistance development is the production of arginase-1 by M2-like macrophages, resulting in depletion of arginine in TME and, consequently, inhibition of T-cells’ function [51]. It was also shown that co-culturing of M2 macrophages with HCC cells resulted in autophagy enhancement in tumor cells and increased resistance to oxaliplatin. Inhibition of autophagy-related 5 homolog (ATG5), a key contributor of autophagy, conversely, reversed the resistance and recovered sensitivity to oxaliplatin [52]. The similar mechanism of chemoresistance development was demonstrated in work [53]: treatment of gastric cancer with 5-fluoruracil (5-FU) was associated with activation of HIF1α signaling and expression of damage-associated molecular patterns, HMGB1, in tumor cells, which, in turn, enhanced macrophage recruiting and their polarization towards M2. M2 TAMs release growth differentiation factor 15 (GDF15), a member of the transforming growth factor beta (TGF-β) family, to stimulate fatty acid oxidation in tumor cells. Therefore, fatty acids are used by tumor cells as a fuel, conferring chemoresistance [53]. Examples of TAM-produced growth factors, contributing to chemotherapy resistance, include TGF-β1, overproduced by M2 TAMs in glioma, which enhance stemness and resistance of glioma cells via the SMAD2/3 signaling pathway [54]; milk-fat globule epidermal growth factor-VIII (MFG-E8), discovered in TAMs of colon cancer and NSCLC, which activates STAT3; and Sonic Hedgehog signaling in tumor cells resulting in increased tumorigenicity and drug resistance [55]. In cisplatin-resistant ovary cancer, overexpression of circular RNA circITGB6 was revealed. circITGB6 indirectly increase the stability of fibroblast growth factor 9 (FGF9) mRNA that enhances M2 macrophage polarization and is associated with supporting resistance to cisplatin [56]. Cytokines, released by TAMs, can also activate signaling pathways, involved in proliferation and chemotherapy resistance in cancer cells: TAM-derived CCL5 induced activation of signaling pathway STAT3/Nanog in prostate cancer cells, which was related with enhanced resistance to paclitaxel. Addition of STAT3 inhibitor to paclitaxel therapy resulted in significant mitigation of chemotherapy resistance in mouse model of prostate cancer and prolonged survival of animals [57]. In lung cancer resistant to cisplatin and doxorubicin, overexpression of IL-34 by tumor cells enhanced survival of chemo-resistant cells via activation of CSF1R-mediated AKT signaling and promoted TAM polarization towards M2 enhancing macrophage immunosuppressive function through CCAAT/enhancer-binding protein β-mediated mechanism [58]. In breast cancer TAMs-secreted CCL2 activated PI3K/AKT/mTOR signaling, associated with tamoxifen resistance [59]. The same signaling pathway associated with upregulation of anti-apoptotic protein Bcl-2, downregulation of pro-apoptotic protein Bax, and increase in resistance to 5-FU, was activated in gastric cancer cells under the effect of CXCL5, produced by TAMs. Moreover, CXCL5 promoted recruiting of monocytes and their polarization towards M2, forming the feedback loop [48]. In general, upregulation of anti-apoptotic proteins and, consequently, prevention of apoptosis, is another mechanism of chemoresistance development, involving TAMs. IL-10, produced by TAMs, enhanced paclitaxel resistance via activation of STAT3 and upregulation of Bcl-2 in breast cancer cells [49]. In cisplatin-resistant gastric cancer, production of miR-21 by TAMs was shown which decreased PTEN activation, and enhanced activation of the PI3K/AKT pathway and production of Bcl-2, therefore, decreasing apoptosis rate and cisplatin sensitivity of gastric cancer cells [60].

**Figure 3:**
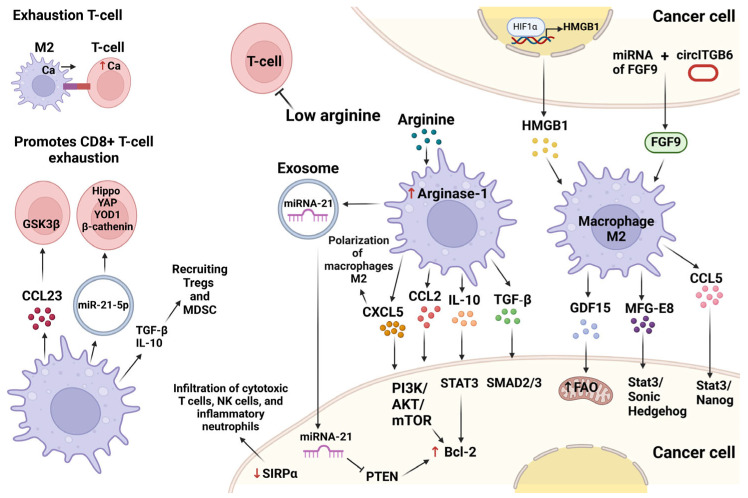
Schematic representation of signaling pathways associated with chemoresistance development and immune escape of cancer cells (see Section 4.2 and Section 4.3 for more details). Figure was created with BioRender.com.

Signaling pathways associated with the development of chemotherapy resistance in cancer cells are summarized in Figure 3.

### 4.3. TAMs Promote Tumor Immune Escape

Tumor immune escape is closely related to the therapy resistance and it may be realized via the following mechanisms, involving TAMs: attraction of immunosupressive cells to the TME, (via CCL22 [61], TGF-β and IL-10 [62]) stimulation of effector T-cells exhaustion and dysfunction [63,64,65], expression of immunosuppressive molecules or their receptors, including programmed death-ligand 1/programmed death-1 (PD-L1/PD-1), LAG-3 and CTLA4, or ‘do-not eat me’ signal (CD47) on the surface of the tumor cells [62] that can inhibit the activation of effector T-cells. A number of research groups have demonstrated the presence of spatiotemporal correlation between effector CD8^+^ T-cells and tumor-associated macrophages, moreover, expression levels of M2 markers (CD163^+^, CD206^+^, etc.) closely correlated with T-cell exhaustion—condition, characterized by dysfunction of effector cells and marked by the upregulation of inhibitory receptors (such as programmed cell death-1 (PD-1), T cell immunoglobulin and mucin domain-3 protein (TIM-3), etc.) [66,67]. Therefore, TAMs play an important role in stimulation of CD8^+^ T-cell exhaustion. Data of microscopy analysis and calcium imaging showed that TAMs form long-lasting contacts with CD8^+^ cells, stimulating weak prolonged Ca^2+^ flux in CD8^+^ T-cells, which fail to support their proliferation, but can result in their exhaustion [68]. CD8^+^ T-cells exhaustion could also occur under the effect of different factors, produced by TAMs, in particular, under the effect of TAM-derived CCL23. Treatment of CD8^+^ cells with CCL23 in in vitro experiments resulted in a dose-dependent increase in exhaustion markers. This CCL23 effect was mediated by glycogen synthase kinase 3β signaling [63]. TAM-derived extracellular vesicles contain overexpressed miR-21-5p, which promotes CD8^+^ T-cell exhaustion via a pathway, including the components of the Hippo signaling pathway, YAP and YOD1, and β-cathenin [65]. Another example of TAM-mediated immunosuppression M2-like TAMs, activated under the effect of radiotherapy-induced damage-associated molecular patterns, created an immunosuppressive niche by releasing TGF-β and IL-10 and recruiting regulatory T-cells and myeloid-derived suppressor cells into TME. However, knockout of signal-regulatory protein α (SIRPα)—regulator that inhibits phagocytosis of tumor cells via interacting with the self-recognition marker CD47 (the so-called ‘do not eat me’ signal)—resulted in infiltration of cytotoxic T cells, NK cells, and inflammatory neutrophils, therefore, enhancing radiotherapy efficiency and promoting tumor elimination [62].

### 4.4. TAMs Promote EMT and Metastasis

M2-like macrophages release into TME large amounts of serine proteases, cathepsins [69], and metalloproteases [70], as well as pro-angiogenic factors such as VEGF-A and VEGF-C [71]. Proteases released into the tumor microenvironment destroy intercellular contacts and cleave the components of the extracellular matrix, thereby promoting cell migration and, as a result, tumor metastasis. The release of proangiogenic factors into the extracellular space enhances angiogenesis and increases vascular permeability, facilitating the migration of tumor cells and tumor metastasis [72]. In particular, TAM-derived cathepsin B, a papain-like cysteine protease, is able to cleave components of the extracellular matrix such as laminin, collagen V, collagen I, cell adhesion molecules (E-cadherin) and degrade tight cell junctions, thereby promoting migration, epithelial-mesenchymal transition of cells, and also metastasis [73,74].

Data have also been obtained indicating the release of certain signaling molecules by TAMs that trigger EMT-related pathways in cancer cells. In particular, analysis of data obtained from patients with metastatic and non-metastatic colorectal cancer as well as data obtained in animal models showed that TAMs overproduce TGF-β, which contribute to EMT by activation of the Smad2,3-4/Snail/E-cadherin pathway [75]. Another example of stemness and migration under the effect of TGF-β was shown in work [54]: in glioma cells, TAM-derived TGF-β1 upregulated EMT markers (vimentin and N-cadherin) via the Smad2/3 pathway. In in vitro experiments with co-cultured TAMs and hepatocellular carcinoma cells it was shown that M2-like cells produce IL-8 which upregulates EMT markers in HCC cells via the JAK2/STAT3 pathway [76].

## 5. Non-Targeted Cancer Therapy Related to TAMs

Therefore, we can see that TAMs are involved in all pathological processes leading to cancer progression. In this regard, a number of clinical trials of the drugs affecting TAMs are currently being conducted. Some studies have already been completed, and results are available. Strategies to impact the TAMs include the depletion of TAMs (in particular, inhibition of signal molecules, that recruit monocytes and macrophages), re-education of TAMs towards M1-like phenotype, and blocking the activity of TAM-derived molecules with immune-suppressive functions (such as TGF-β and IL-10). In this review we summarized novel data about various non-targeted interventions for tackling TAMs, which have been tested in recent and ongoing clinical trials.

Conventional option for TAM depletion is the inhibition of CSF-1/CSF-1R recruiting axis. Among the novel CSF-1R inhibitors emactuzumab, recombinant, humanized monoclonal antibody against CSF-1R, was tested in phase 1 trials in patients with urothelial bladder cancer (NCT01494688) [77] and diffuse-type tenosynovial giant cell tumors (dTGGT, NCT02323191) [78]. Treatment of patients with dTGGT with emactuzumab resulted in the pronounced response associated with symptomatic improvement and a manageable safety profile [78]. In both studies emactuzumab treatment resulted in a significant decrease in M2-like TAMs and increased the density of activated CD8^+^ T-cells. However, in comparison with paclitaxel monotherapy, the combination of paclitaxel and emactuzumab failed to increase anticancer activity of therapy in patients with advanced solid tumors. Moreover, no patients on emactuzumab monotherapy showed an objective response [79]. In this case the anticancer activity of emactuzumab is limited only by tenosynovial giant cell tumor indication [78,80]. Anti-CSF-1 mAb, lacnotuzumab, was shown to enhance CD8^+^ T-cell tumor infiltration and sensitivity to paclitaxel in preclinical experiments and xenograft models. However, in a randomized phase 2 clinical trial of lacnotuzumab in combination with gemcitabine and carboplatin versus gem-carbo combination therapy in advanced triple-negative breast cancer, both treatments showed comparable outcomes [81]. A small molecule inhibitor of CCR2 (CCR2-CCL2 signaling axis), PF-04136309, was tested in a phase 1 trial (NCT01413022) in combination with FOLFIRINOX (combination of oxaliplatin, irinotecan, leucovorin, and fluorouracil) in patients with borderline resectable and locally advanced pancreatic adenocarcinoma. In this study, the addition of PF-04136309 to FOLFIRINOX did not enhance toxicity of therapy, but resulted in reduction in the TAM infiltrate, increased the amount of CD8^+^ and CD4^+^ cells, reduced the presence of Tregs, and induced an endogenous anti-tumor immune response [82].

Multikinase inhibitors, such as sorafenib (inhibitor of RAF/MEK/ERK pathway and receptor tyrosine kinases) [83] and lenvatinib (inhibitor of various kinase receptors including VEGFR1–3, fibroblast growth factor receptors 1–4, platelet-derived growth factor receptor α etc.) [84] are FDA approved drugs as a first-line therapy for advanced cancer, such as unresectable HCC [85]. It was demonstrated that lenvatinib modulates TAM activity: RNA sequencing results showed that lenvatinib treatment resulted in increased production of CXCL9 by TAMs and recruitment of CD8^+^ cells. However, the addition of lenvatinib to ICI pembrolizumab failed to show the synergy in anticancer activity, probably due to entrapment of CD8^+^ cells in tumor stroma (NCT03713593) [84].

Downregulation of CD47, checkpoint expressed in monocytes, macrophages, dendritic cells and neutrophils, is another mechanism that results in macrophage reprogramming towards M1. Bromonitrozidine (RRx-001) is an inhibitor of inflammasome component NLRP3, which also inhibits CD47 and showed preliminary anticancer activity in combination with ICI nivolumab. (NCT02518958) [86]. RRx-001 was also evaluated in phase 2 trials as an adjuvant therapy of advanced colorectal cancer (NCT02096354) and metastatic brain cancer (NCT02215512), but results of these trials are not available yet.

Another tool for TAM reprogramming towards M1-like phenotype is activation of CD40, a member of the TNF receptor superfamily, which is expressed on myeloid cells, including macrophages. Selicremumab, agonistic monoclonal antibody (mAb) to CD40, showed promising results along with acceptable toxicity. In an open phase I clinical trial (NCT02588443), treatment with selicrelumab in combination with gemcitabine and nab-paclitaxel resulted in T-cell enrichment and a decrease in M2 macrophages in TME in comparison with chemotherapy alone [87]. Another agonistic antibody to CD40, ABBV-428, targeted on tumor antigen mesothelin, was tested on patients with advanced mesothelioma and ovarian cancer in a phase I clinical trial (NCT02955251); however, its anticancer activity was minimal [88].

Concerning the downregulation of immunosuppressive molecule production, it was shown that treatment with apatinib, a small molecule inhibitor of VEGFR2 approved by National Medical Products Administration (NMPA) of China as a third-line therapy of advanced gastric cancer, significantly improved overall survival and progression-free survival in comparison with placebo in a phase 3 trial [89]. Apatinib indirectly decreased the level of TGF-β, produced by TAMs, that could correlate with the anticancer activity of the drug [90]. A number of phase 2 clinical trials were completed in China to study the efficiency of apatinib in advanced tumors (such as platinum resistant recurrent ovarian cancer (NCT03587129), advanced colorectal cancer (NCT01531777), non-triple-negative metastatic breast cancer (NCT01653561)). However, the results of these trials are still not available.

Bintrafusp alpha is a first-in-class bifunctional fusion protein targeting both TGF-β and PD-L1. In phase 1, an open-label clinical trial in patients with NSCLC, the best outcome was the confirmed partial treatment response; that was achieved in 27.8% of ICI-naïve patients and 0% of ICI-experienced patients with NSCLC (NCT02517398). [91] In this study bintrafusp alpha decreased the M2/M1 ratio in responders and increased this ratio in non-responders; that highlights the role of M2 macrophages in tumor resistance to immune therapy. In a phase 3 clinical trial of bintrafusp alfa versus pembrolizumab in patients with treatment-naïve advanced NSCLC, first-line treatment with bintrafusp alfa failed to demonstrate superior efficacy compared with pembrolizumab.

An innovative approach is the development of chimeric antigen receptor macrophage therapy (CAR-M). Use of CAR-T cell therapy is already established in clinical practice nowadays. But natural phagocytosis capability of macrophages provides an outstanding opportunity to construct CAR-Ms targeted on tumor antigens. One example of such therapy is the construction of CAR-Ms that target HER2 and CD47 on the surface of tumor cells. The resulting macrophages were shown to phagocytose ovarian cancer cells in in vitro and in vivo experiments. Moreover, activation of CD8^+^ T-cells was demonstrated [92]. At the time of writing this review, patient enrollment in phase 1 clinical trials of CAR-M therapy of HER2-overexpressing solid tumors starts (NCT04660929, NCT06224738).

In conclusion, the efficiency of the existing cancer treatment tools is not optimal. It is necessary to narrow indications for treatment and define the target population of patients that could benefit from the use of these therapeutic options. There still exists the unmet need in enhancement of anti-cancer armamentarium. In particular, methods of nanomedicine could enable scientists an opportunity to achieve a more selective effect on M2-like TAMs and provide controlled release of the drug to mitigate adverse events. In the next section we will briefly discuss currently ongoing studies in nanotechnology and nanomedicine aimed at targeting TAMs.

**Figure 4:**
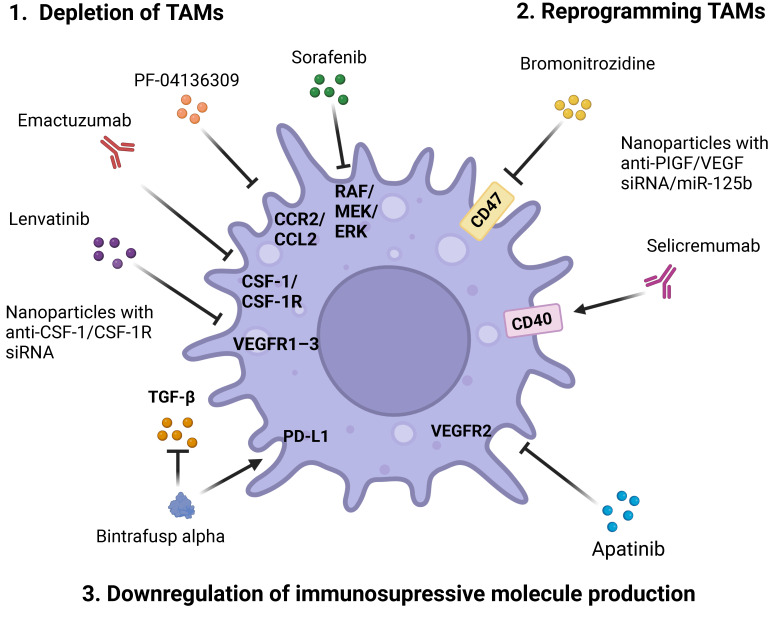
Therapeutic options aimed at targeting TAMs (see Section 5 and Section 6 for more details). Figure was created using BioRender.com.

Some of the therapy options are schematically represented in Figure 4.

## 6. Nanotherapy Options Aimed at Targeting TAMs

### 6.1. TAM Reprogramming to Prevent Tumor Angiogenesis

As previously mentioned, the formation of new blood vessels in the TME is stimulated by hypoxia and is closely linked to tumor progression and metastasis. Hence, antiangiogenic therapy holds significant importance in cancer treatment. Presently, drugs like sorafenib, sunitinib, and regorafenib are utilized for antiangiogenic purposes. Specifically, sorafenib diminishes the expression of key factors such as vascular endothelial growth factor receptor (VEGFR), platelet-derived growth factor receptor (PDGFR-β), and hepatocyte growth factor receptor (HGFR) [93,94,95]. However, disadvantages of such drugs include rapid development of drug resistance [94,96], enhancement of hypoxia in TME, and risk of oncogene expression activation [95]. Hence, there is a pressing need to explore alternative anti-angiogenic therapies, particularly those targeting TAMs, as they are the primary producers of VEGF-A, VEGF-C [10], MMP-7, and MMP-9 [70].

In an effort to enhance sensitivity to sorafenib, nanoparticles were developed containing sorafenib and a modified TAM repolarization agent, resiquimod. These nanoparticles were formulated using a pH-responsive block copolymer, methoxyl-PEG-Dlink_m_–PLGA. pH-triggered PEG detachment enabled NPs the opportunity to efficiently accumulate in HCC cells and in TAMs after intravenous injection to mice. Systemic treatment with nanoparticles resulted in TAMs’ repolarization towards M1, suppression of VEGF and, consequently angiogenesis, and reduction of tumor growth [95]. A successful attempt to downregulate VEGF, an angiogenic factor, responsible for tumor neovascularization and lymphangiogenesis, was made in a lung adenocarcinoma mouse model. Gold core NPs, covered with thiolated PEG-COOH polymer, thiolated anti-VEGF siRNA, and M2 peptide, targeting the M2 MARCO scavenger receptor, were instilled intratracheally to mice resulting in efficient decrease in VEGF expression level in lungs and ~95% reduction of TAMs that mediated a delay in lung cancer progression and survival increase [9]. Another attempt to downregulate VEGF along with placental growth factor (PIGF) in M2 macrophages was made [39]. PEGylated chitosan-based nanoparticles coated with mannose residues and containing anti-VEGF and anti-PIGF siRNAs were efficiently targeted and provided simultaneous downregulation of VEGF and PIGF, which resulted in the remodeling of the tumor microenvironment with an enhancement of the antitumor immune response and, as a consequence, decreased tumor growth and decreased incidence of lung metastases formation.

Reprogramming of TAMs in combination with paclitaxel chemotherapy was attempted in a non-small cell lung cancer model [97] and in a syngeneic ID8-VEGF ovarian cancer mouse model [98] using intra-peritoneal injection of hyaluronic acid-PEI NPs containing regulatory miR-125b. HA in NPs was used as a tool to target constructed NPs to CD44 receptor, expressed on the surface of TAMs. Treatment with NPs in combination with paclitaxel resulted in significant reduction of ascites volume, a decrease in VEGF level and an increase in the M1 (CD80^+^ cells)/M2 (CD206^+^ cells) TAMs ratio in comparison with NP or paclitaxel treatment alone.

G5-dendrimer nanoparticles loaded with methotrexate were used to target folate receptor-2 on the surface of TAMs in ovarian cancer [10]. Intraperitoneal injection of the obtained G5-MTX nanoparticles to mice with ID8-VEGF ovarian tumors resulted in efficient depletion of TAMs, significant reduction of microvessel density, decrease in expression levels of VEGF-A, VEGF-C, mitigated resistance to anti-VEGF therapy, and improved survival.

In another study [99], exosomes derived from macrophages were electroporated with extremely small iron oxide nanoparticles (ESIONPs@EXO) which induce ferroptosis. It was shown that treatment with ESIONPs@EXO resulted in M2 to M1 reprogramming, significantly inhibited vessel formation, reduced endothelial cell sprouting, and suppressed pathological angiogenesis in vivo via VEGF-independent mechanism in the ocular melanoma model, also prohibiting tumor cell proliferation and migration.

Another innovative nanoformulation was applied to combat angiogenesis in the melanoma model: IL-13-conjugated long-circulating liposomes, loaded with the chemotherapy drug simvaststin (IL-13-LCL-SIM) and PEG-coated extracellular vesicles derived from melanoma cells and loaded by doxorubicin (PEG-EV-DOX), were used in combination and demonstrated effective targeting both to TAMs and melanoma cells and strong suppression of a number of pro-angiogenic factors (VEGF, bFGF, MCP1 and endothelial cell proliferation marker CD31), resulting in hindered tumor growth [100]. A brief description of all nanotherapy approaches used for TAM reprogramming in the context of cancer therapy is summarized in Table 1.

### 6.2. TAM Reprogramming to Increase Conventional Therapy Effect and Overcome Therapy Resistance

It was previously discussed that an increased amount of M2 macrophages in the tumor microenvironment may contribute to chemotherapy resistance and immune escape. Nevertheless, some novel scientific data show that TAM reprogramming can result in increased efficiency of chemo- and radiotherapy and overcoming therapy resistance. One example of increased efficiency of isotope therapy as a result of TAM reprogramming was shown [101]. In this study, NPs were produced from Ca^2+^ and bisphosphonates and covered by PEG. The pH-dependent degradation of PEG facilitated in the acidic TME induced the release of bisphosphonates, a cost-effective drug known for its ability to deplete the TAMs. Furthermore, the incorporation of ^90m^Tc or ^32^P isotopes to NPs provided an opportunity to visualize a tumor by single-photon emission computed tomography imaging or induce synergistic cancer radioisotope therapy, resulting in mitigation of hypoxia and a significant reduction of breast cancer tumor growth.

One strategy to mitigate the development of therapy resistance involves the simultaneous administration of multiple therapeutic agents. In a recent study [102], co-delivery of CSF1R inhibitor and the inhibitor of Src homology region 2 domain-phosphatase 2 (SHP2)—member of the signaling axis CD47-SIRPα (inhibitor SHP099)—was performed. NPs were produced from co-formulated lipids, mainly phosphatidylcholine, PEGylated, and functionalized with anti-CD206 antibodies to bind mannose receptors on the surface of M2. Intravenous injection created NPs in aggressive 4T1 breast cancer, and B16/F10 melanoma mouse models resulted in enrichment of TME with M1 macrophages and improved efficiency of combination anticancer therapy. Moreover, the authors suggested that combining these novel nanotherapeutics with conventional chemo- or immunotherapy holds promise as a synergistic treatment approach.

Another strategy to enhance therapy efficacy involves elevating drug concentrations within tumor cells and the tumor microenvironment TME through targeted delivery and controlled drug release using nanocarriers. For instance, NPs carrying paclitaxel (PTX) were synthesized using methoxy-poly(ethylene glycol)-poly(lactic acid) as a base and functionalized with cell-penetrating TAT-peptide and tumor-homing LinTT1 peptide, linked via a metalloproteinase-cleavable linker [103]. Introduction of linkers, cleavable by proteases, enriched in acidic TME (i.e., cathepsins, metalloproteases etc.) is a widely used approach in nanotechnology (for more information see review [116]). The resulting nanoparticles (NPs) were efficiently internalized into tumor cells and tumor-associated macrophages (TAMs), achieving a remarkable 90% inhibition of tumor growth in vivo compared to the control PBS-treated group and a 60% inhibition compared to NPs carrying paclitaxel without functionalization. This led to a significant prolongation of survival.

In another therapeutic approach, a peptide hydrogel was formed by combining the pro-apoptotic peptide Smac with a Toll-like receptor TLR7/8 agonist. Systemic injection of Smac-TLR7/8 hydrogel, either alone or in combination with radiotherapy or immunotherapy, activated NF-κB signaling, resulting in a strong shift of the M1/M2 ratio toward M1 macrophages. This activation enhanced the antitumor effects of macrophages, increased the rate of DNA breakage formation, thereby overcoming radiotherapy resistance, and ultimately reduced tumor growth in vivo in the melanoma model [104].

Another therapeutic approach of highly hypoxic tumors was suggested to combine NPs from manganese dioxide MnO_2_, which can produce oxygen to relieve hypoxia and be used as a contrast agent for MRI tumor imaging and hyaluronic acid, which is reported to enhance M1 macrophage polarization and increase NP biocompatibility. Mannan was applied as a targeting agent to CD206 mannose receptors of M2. Intravenous injection of the obtained nanoparticles resulted in macrophage reprogramming towards M1 and an increase in the H_2_O_2_ level in TME of breast cancer. Moreover, NPs relieved tumor hypoxia two times according to pimonidazole marker analysis and drastically decreased expression levels of HIF1α and VEGF. This nanosystem for tumor detection and therapy also increased the efficiency of doxorubicin, injected in 6 h after NP [105].

### 6.3. TAM Reprogramming to Prevent Metastasis Formation

The strategies employed to reprogram macrophages have demonstrated efficacy in reducing tumor metastasis. This section presents several examples of the impact of nanotherapeutics-induced macrophage repolarization on cancer metastasis. Hydrazinocurcumin was shown to inhibit STAT3—a key factor in M2 macrophage polarization. Therefore, lyposomal NPs were produced from DOPE, DOPC, cholesterol, DOPE-PEG and hydrazinocurcumin. In addition, legumain inhibitor was incorporated into nanoparticles to inactivate legumain protease, overexpressed by M2 macrophages in order to reprogram them towards M1 [106,117]. The obtained NP efficiently decreased expression levels of STAT3, MMP2, MMP9 and VEGF, inhibited breast cancer growth, prolonged survival of animals, and reduced pulmonary metastasis three times. Another study developed a nanotherapeutic system to tackle highly invasive melanoma. PLGA nanoparticles were covered with polydopamine and loaded with immune-modulatory agent baicalin and melanoma antigen Hgp peptide fragment 25–33. Immune stimulator cytosine-guanosine oligodeoxynucleotide (CpG-ODN) inducing macrophage repolarization towards M1 and peptides M2pep and α-pep for dual targeting of NPs to M2 macrophages were adsorbed on the surface of the NP via linking with polydopamine [107]. Systemically injected NPs were effectively targeted to M2-like TAMs in melanoma tumors, induced efficient macrophage reprogramming, and significantly decreased growth and metastasis of the B16 melanoma tumor [107].

Additionally, an effective strategy to reduce metastasis burden involves the application of nanoparticles for photodynamic immunotherapy coated with a TAM-derived cell membrane. These nanoparticles, comprising rare-earth-upconversion-nanoparticle-based photosensitizer NaYF4:Yb,Er@NaYF4, covered with TAM-derived membrane, possess antigen-homing affinity capacity and immune compatibility, contributing to the depletion of CSF1 secreted by tumor cells and blockade of the interaction between TAMs and cancer cells [108]. Systemic injection of obtained NPs resulted in macrophage reprogramming, and a significant decrease in tumor growth and number of pulmonary metastatic nodules in the 4T1 breast cancer mouse model [108].

### 6.4. Other Strategies of TAM Reprogramming to Restrict Tumor Growth and Increase Survival

Qian et al. (2017) developed lipid nanoparticles carrying anti-CSF-1R siRNA using a dual targeting strategy: they linked together M2-targeting peptide M2pep and ApoA1-mimetic α-helical peptide binding to scavenger receptor B-type on the surface of M2. The resulting nanoparticles were efficiently sequestered by M2 macrophages, depleted the number of TAMs, and decreased the growth of melanoma tumors in vivo [109]. Blockade of the same signaling pathway was obtained in [110] on the mouse model of liver fibrosis. CSF-1R was downregulated in M2 TAMs using nanohydrogel formulation containing Man-P(MEO_3_MA)_18_-b-P(PFPMA)_30_, spermine and triethylamine, and anti-CSF-1 siRNA. Intravenous injection of mannose-functionalized nanohydrogel formulation resulted in efficient accumulation of nanohydrogel in liver area and, in particular, its efficient sequestration by M2 TAMs. The resulting nanoformulation could be successfully applied for immunomodulatory treatment of liver fibrosis and cancer.

Liu et al. (2018) created ROS-inducing polypeptide micelles comprising zinc protoporphyrin IX, covered by poly(L-lysine)-b-poly(ethyleneglycol), containing TLR3 agonist Poly I:C, bound via electrostatic adsorption. NPs were functionalized with galactose in order to target them to TAMs via galactose-specific C-type lectin. Intratumoral injection of the obtained NP to tumor-bearing mice resulted in effective sequestration of polypeptide micelles by TAMs and TAM reprogramming into M1 associated with an increase in the ROS level and downregulation of STAT3, followed by apoptosis of tumor tissues [111].

Wang et al. (2020) performed systemic delivery of miR-99b, which is responsible for regulation of myeloid cell differentiation and macrophage activation to TAMs of mice with highly aggressive hepatocellular carcinoma (HCC) and Lewis lung cancer. miR-99b was encapsulated in nanoparticles comprised of cationic konjac polysaccharide and PEG-His-modified alginate. miR-99b downregulated expression of mTOR, its downstream transcription factor IRF4, participating in M2 activation, and kB-Ras2 that resulted in activation of NF-κB signaling and reprogramming of M2 macrophages into M1. It was also shown that miR-99b did not affect transcription factor STAT3. Reprogramming was associated with increased expression levels of MHCII in macrophages; increased levels of inflammatory cytokines, such as TNF-α, IL-6, and IL-12; enhancement of macrophage capability of phagocytosis and antigen presentation; and, consequently, reduction of tumor growth [112].

D’Urso et al. (2023) provided delivery of siRNA targeting protein kinase R-like ER kinase (PERK)—the main factor of unfolded protein response (UPR) in endoplasmic reticulum of TAM—in primary murine macrophages using Fe_3_O_4_ magnetic nanoparticles, covered with polydopamine which can bind siRNAs. Suppression of PERK resulted in downregulation of other downstream effectors of the PERK pathway, contributing to UPR (ATF4 and CHOP), and a decrease in level of M2 markers (in particular CD206) and an increase in M1 markers (CD86 and inflammatory cytokines). Therefore, authors concluded that modulation of UPR resulted in reprogramming of M2 to M1 [113].

In the work [114], the authors linked together tetrahedral framework nucleic acid to cytosine-guanosine oligodeoxynucleotide (CpG ODN), which is an agonist of TLR9, and anti-PI3Kγ (phosphatidylinositol triphosphate kinase) siRNA. Obtained NPs enabled TLR9 activation in TAMs that resulted in activation of NF-κB signaling cascade which is related to activation of inflammatory gene transcription (M1 phenotype). Simultaneous downregulation of PI3Kγ enhanced this effect. Therefore, systemic injection of NPs to tumor-bearing mice resulted in reprogramming of TAMs toward M1, tumor growth delay, and prolonged survival.

An elegant approach of immune checkpoint blockade therapy was used in work in [115]. In this work extracellular vesicles were obtained from M1 macrophages, overexpressing fusogenic glycoprotein of vesicular stomatitis virus (VSV) and electroporated with anti-PD-L1-siRNA. The presence of VSV glycoprotein on the surface of extracellular vesicles allowed them to bypass endocytosis pathways and release siRNA into cytoplasm. Intravenous injection of the resulting extracellular vesicles to CT26 tumor-bearing mice (colon carcinoma) resulted in downregulation of PD-L1 in tumor tissues, disruption of PD-1-PD-L1 interaction, an increased amount of CD8^+^ cells, and an elevated M1/M2 ratio, mediated by an increase in the IFNγ level, and, consequently, a significantly increased survival of mice.

## 7. Conclusions and Future Perspectives

As discussed in this review, tumor-associated macrophages play an important role in every single aspect of tumor progression, as well as in chemotherapy and immunotherapy resistance. The existing therapy options aimed at combatting TAMs demonstrate limited clinical efficiency and require careful selection of the target patient population and indications for the use of these drugs. Rapidly developing nanotechnology approaches are designed to satisfy the unmet need for targeted delivery of the drugs, decreased adverse events, and more selective effects on tumor-supporting M2-like macrophages, therefore, overcoming limitations inherent in conventional therapies. As for the limitations of nanotechnology, the more sophisticated surface modifications of nanoparticles are created to increase their circulation time and targeting efficiency. From the targeted delivery of therapeutic agents to TAMs to the modulation of complex signaling pathways within the tumor microenvironment, these approaches offer new avenues for improving patient outcomes. Moreover, the synergy between nanotechnology and immunotherapy opens new possibilities for personalized and combination therapies, paving the way for more effective and tailored cancer treatments. As research in this field continues to evolve, the integration of these innovative strategies into clinical practice holds great promise for revolutionizing cancer care and improving patient survival and quality of life.

## Figures and Tables

**Table 1:** Cancer nanotherapy options for TAM reprogramming.

NP Composition	Active Payload	Target on TAMs	Targeting Moiety	Cancer Type/Effect	Ref.
Methoxyl-PEG-Dlinkm–PLGA	Sorafenib and resiquimod			HCC/suppression of VEGF, angiogenesis, tumor growth	[95]
PEGylated gold	Anti-VEGF siRNA	MARCO	M2 peptide	Lung adenocarcinoma/suppression of VEGF, ~95% reduction of TAMs, delay in lung cancer progression, survival increase	[9]
PEGylated trimethyl chitosan with citraconic anhydride grafted poly (allylamine hydrochloride)	Anti-VEGF and anti-PIGF siRNAs	CD206	Mannose	BC/suppression of VEGF and PIGF, decrease in tumor growth and lung metastasis	[39]
HA-PEI	miR-125b	CD44	HA	NSCLC, ovarian cancer/ suppression of VEGF, increase in M1/M2 ratio, reduction of ascites volume	[97,98]
G5-dendrimer	Methotrexate	Folate receptor-2	Methotrexate	Ovarian cancer/suppression of VEGF-A, VEGF-C, suppressed resistance to anti-VEGF therapy and improved survival	[10]
Exosomes from TAMs incorporated with extremely small iron oxide nanoparticles	Ferroptosis	TAM membrane	TAM membrane	Ocular melanoma/suppressed vessel formation, endothelial cell sprouting, angiogenesis, tumor aggressiveness	[99]
IL-13-conjugated long-circulating liposomes and PEGylated extracellular vesicles	Simvaststin, doxorubicin	IL-13 receptors	IL-13	Melanoma/suppression of VEGF, bFGF, MCP1, CD31, decrease in tumor growth	[100]
PEGylated calcium NP	Bisphosphonates, 32P isotopes			BC/increased efficiency of isotope therapy, significant reduction of hypoxia and tumor growth	[101]
Lipids, predominantly phosphatidyl-choline + PEG	Inhibitors of CSF1R and SHP2	CD206	Antibodies to CD206	BC, melanoma/increase in M1/M2 ratio, improved therapy efficiency	[102]
Methoxy-PEG-poly(lactic acid)	Paclitaxel		Tumor-homing LinTT1 peptide	Lung cancer/improved therapy efficiency, 90% inhibition of tumor growth, significant prolongation of survival	[103]
Peptide hydrogel	Pro-apoptotic peptide Smac and Toll-like receptor TLR7/8 agonist			Melanoma/increase in M1/M2 ratio, overcoming of radiotherapy resistance	[104]
MnO2, covered with hyaluronic acid	Hyaluronic acid	CD206	Mannan	BC/suppression of HIF1α and VEGF, increased the efficiency of doxorubicin	[105]
DOPE, DOPC, cholesterol, DOPE-PEG	Hydrazino-curcumin and legumain inhibitor			BC/suppression of STAT3, MMP2, MMP9 and VEGF, delayed tumor growth, prolonged survival, reduced metastasis incidence	[106]
PLGA nanoparticles covered with polydopamine	Baicalin, melanoma antigen Hgp peptide and CpG-ODN	MARCO, scavenger receptor B type 1	M2pep and α-pep	Melanoma/increase in M1/M2 ratio, significant reduction of tumor growth and metastasis	[107]
NaYF4:Yb,Er@NaYF4	Photodynamic immunotherapy		TAM-derived cell membrane	BC/macrophage reprogramming, significant decrease in tumor growth and number of pulmonary metastatic nodules	[108]
Phospholipids	Anti-CSF-1R siRNA	MARCO, scavenger receptor B-type	M2pep + α-helical peptide	Melanoma/depletion of TAMs, decreased tumor growth	[109]
Man-P(MEO3MA)18-b-P(PFPMA)30, spermine, triethylamine	Anti-CSF-1 siRNA	CD206	Mannose	liver cancer/efficient targeting to M2-like macrophages	[110]
Poly(L-lysine)-b-PEG	TLR3 agonist Poly I:C	Galactose-specific C-type lectin	Galactose	Melanoma/increased M1/M2 ratio, ROS level, downregulation of STAT3, apoptosis of tumor tissues	[111]
Cationic konjac polysaccharide and PEG-His-modified alginate	miR-99b			HCC, Lewis lung cancer/increased M1/M2 ratio, increased phagocytosis and antigen presentation ability of macrophages, reduction of tumor growth	[112]
Fe3O4 NP, covered with polydopamine	Anti-PERK siRNA			Macrophages derived from murine peritoneal exudate/inhibition of unfolded protein response, increased M1/M2 ratio	[113]
Tetrahedral framework nucleic acid + CpG ODN	Agonist of TLR9 and anti-PI3Kγ siRNA			BC/increased M1/M2 ratio, delayed tumor growth, prolonged survival	[114]
M1-derived extracellular vesicles covered by fusogenic glycoprotein of VSV	anti-PD-L1 siRNA			Colon carcinoma/ downregulation of PD-L1 in tumor, increased M1/M2 ratio, increased IFNγ level, significantly increased survival	[115]

## List of Abbreviations

TAM	Tumor-associating macrophage
TME	Tumor microenvironment
IL	Interleukin
CCL	CC chemokine ligand
MHC	Major histocompatibility complex
ROS	Reactive oxygen species
EMT	Epithelial-mesenchymal transition
mAb	Monoclonal antibody
ICI	Immune checkpoint inhibitor
NP	Nanoparticle
PEG	Polyethylene glycol
PLGA	Poly lactic-co-glycolic acid
BC	Breast cancer
CRC	Colorectal cancer
NSCLC	Non-small cell lung cancer

## References

[1] Rasheed A., Rayner K.J. (2021). Macrophage responses to environmental stimuli during homeostasis and disease. Endocr. Rev..

[2] Cox N., Pokrovskii M., Vicario R., Geissmann F. (2021). Origins, Biology, and Diseases of Tissue Macrophages. Annu. Rev. Immunol..

[3] Zheng H., Peng X., Yang S., Li X., Huang M., Wei S., Zhang S., He G., Liu J., Fan Q. (2023). Targeting tumor-associated macrophages in hepatocellular carcinoma: Biology, strategy, and immunotherapy. Cell Death Discov..

[4] Chen P., Zhang X., Venosa A., Lee I.H., Myers D., Holloway J.A., Prud’homme R.K., Gao D., Szekely Z., Laskin J.D. (2020). A novel bivalent mannosylated targeting ligand displayed on nanoparticles selectively targets anti-inflammatory M2 macrophages. Pharmaceutics.

[5] Yang L., Zhang Y. (2017). Tumor-associated macrophages: From basic research to clinical application. J. Hematol. Oncol..

[6] Qin R., Ren W., Ya G., Wang B., He J., Ren S., Jiang L., Zhao S. (2023). Role of chemokines in the crosstalk between tumor and tumor-associated macrophages. Clin. Exp. Med..

[7] Truxova I., Cibula D., Spisek R., Fucikova J. (2023). Targeting tumor-associated macrophages for successful immunotherapy of ovarian carcinoma. J. Immunother. Cancer.

[8] Olmsted-Davis E., Mejia J., Salisbury E., Gugala Z., Davis A.R. (2021). A population of M2 macrophages associated with bone formation. Front. Immunol..

[9] Conde J., Bao C., Tan Y., Cui D., Edelman E.R., Azevedo H.S., Byrne H.J., Artzi N., Tian F. (2015). Dual targeted immunotherapy via in vivo delivery of biohybrid RNAi-peptide nanoparticles to tumor-associated macrophages and cancer cells. Adv. Funct. Mater..

[10] Penn C.A., Yang K., Zong H., Lim J.-Y., Cole A., Yang D., Baker J., Goonewardena S.N., Buckanovich R.J. (2018). Therapeutic impact of nanoparticle therapy targeting tumor-associated macrophages. Mol. Cancer Ther..

[11] Pan Y., Yu Y., Wang X., Zhang T. (2020). Tumor-associated macrophages in tumor immunity. Front. Immunol..

[12] Zhang Q., Cheng S., Wang Y., Wang M., Lu Y., Wen Z., Ge Y., Ma Q., Chen Y., Zhang Y. (2021). Interrogation of the microenvironmental landscape in spinal ependymomas reveals dual functions of tumor-associated macrophages. Nat. Commun..

[13] Qian Y., Yin Y., Zheng X., Liu Z., Wang X. (2024). Metabolic regulation of tumor-associated macrophage heterogeneity: Insights into the tumor microenvironment and immunotherapeutic opportunities. Biomark. Res..

[14] Zeng W., Liu P., Pan W., Singh S.R., Wei Y. (2015). Hypoxia and hypoxia inducible factors in tumor metabolism. Cancer Lett..

[15] Shobaki N., Sato Y., Suzuki Y., Okabe N., Harashima H. (2020). Manipulating the function of tumor-associated macrophages by siRNA-loaded lipid nanoparticles for cancer immunotherapy. J. Control. Release.

[16] Hou S.-M., Lin C.-Y., Fong Y.-C., Tang C.-H. (2023). Hypoxia-regulated exosomes mediate M2 macrophage polarization and promote metastasis in chondrosarcoma. Aging.

[17] Zhao S., Mi Y., Guan B., Zheng B., Wei P., Gu Y., Zhang Z., Cai S., Xu Y., Li X. (2020). Tumor-derived exosomal miR-934 induces macrophage M2 polarization to promote liver metastasis of colorectal cancer. J. Hematol. Oncol..

[18] Zhang P., Xia Q., Liu L., Li S., Dong L. (2020). Current opinion on molecular characterization for GBM classification in guiding clinical diagnosis, prognosis, and therapy. Front. Mol. Biosci..

[19] Qiu W., Guo X., Li B., Wang J., Qi Y., Chen Z., Zhao R., Deng L., Qian M., Wang S. (2021). Exosomal miR-1246 from glioma patient body fluids drives the differentiation and activation of myeloid-derived suppressor cells. Mol. Ther..

[20] Xu H., Li M., Pan Z., Zhang Z., Gao Z., Zhao R., Li B., Qi Y., Qiu W., Guo Q. (2022). miR-3184-3p enriched in cerebrospinal fluid exosomes contributes to progression of glioma and promotes M2-like macrophage polarization. Cancer Sci..

[21] Xu J., Zhang J., Zhang Z., Gao Z., Qi Y., Qiu W., Pan Z., Guo Q., Li B., Zhao S. (2021). Hypoxic glioma-derived exosomes promote M2-like macrophage polarization by enhancing autophagy induction. Cell Death Dis..

[22] Lu Q., Wang X., Zhu J., Fei X., Chen H., Li C. (2020). Hypoxic tumor-derived exosomal Circ0048117 facilitates M2 macrophage polarization acting as miR-140 sponge in esophageal squamous cell carcinoma. OncoTargets Ther..

[23] Lin F., Yin H., Li X., Zhu G., He W., Gou X. (2019). Bladder cancer cell-secreted exosomal miR-21 activates the PI3K/AKT pathway in macrophages to promote cancer progression. Int. J. Oncol..

[24] Song J., Yang P., Li X., Zhu X., Liu M., Duan X., Liu R. (2021). Esophageal cancer-derived extracellular vesicle miR-21-5p contributes to EMT of ESCC cells by disorganizing macrophage polarization. Cancers.

[25] Zhou S., Lan Y., Li Y., Li Z., Pu J., Wei L. (2022). Hypoxic tumor-derived exosomes induce M2 macrophage polarization via PKM2/AMPK to promote lung cancer progression. Cell Transplant..

[26] Zhang C., Cheng W., Yang T., Fang H., Zhang R. (2023). Lactate secreted by esophageal cancer cells induces M2 macrophage polarization via the AKT/ERK pathway. Thorac. Cancer.

[27] Holm E., Hagmüller E., Staedt U., Schlickeiser G., Günther H.J., Leweling H., Tokus M., Kollmar H.B. (1995). Substrate balances across colonic carcinomas in humans. Cancer Res..

[28] Bogdanov A., Bogdanov A., Chubenko V., Volkov N., Moiseenko F., Moiseyenko V. (2022). Tumor acidity: From hallmark of cancer to target of treatment. Front. Oncol..

[29] Zhou H., Yao J., Zhong Z., Wei H., He Y., Li W., Hu K. (2023). Lactate-Induced CCL8 in Tumor-Associated Macrophages Accelerates the Progression of Colorectal Cancer through the CCL8/CCR5/mTORC1 Axis. Cancers.

[30] Lian G., Chen S., Ouyang M., Li F., Chen L., Yang J. (2019). Colon cancer cell secretes EGF to promote M2 polarization of TAM through EGFR/PI3K/AKT/mTOR pathway. Technol. Cancer Res. Treat..

[31] Zhang A., Xu Y., Xu H., Ren J., Meng T., Ni Y., Zhu Q., Zhang W.-B., Pan Y.-B., Jin J. (2021). Lactate-induced M2 polarization of tumor-associated macrophages promotes the invasion of pituitary adenoma by secreting CCL17. Theranostics.

[32] Wu J.-Y., Huang T.-W., Hsieh Y.-T., Wang Y.-F., Yen C.-C., Lee G.-L., Yeh C.-C., Peng Y.-J., Kuo Y.-Y., Wen H.-T. (2020). Cancer-derived succinate promotes macrophage polarization and cancer metastasis via succinate receptor. Mol. Cell.

[33] Colegio O.R., Chu N.-Q., Szabo A.L., Chu T., Rhebergen A.M., Jairam V., Cyrus N., Brokowski C.E., Eisenbarth S.C., Phillips G.M. (2014). Functional polarization of tumour-associated macrophages by tumour-derived lactic acid. Nature.

[34] Gómez V., Eykyn T.R., Mustapha R., Flores-Borja F., Male V., Barber P.R., Patsialou A., Green R., Panagaki F., Li C.W. (2020). Breast cancer–associated macrophages promote tumorigenesis by suppressing succinate dehydrogenase in tumor cells. Sci. Signal..

[35] Feng R., Morine Y., Ikemoto T., Imura S., Iwahashi S., Saito Y., Shimada M. (2018). Nrf2 activation drive macrophages polarization and cancer cell epithelial-mesenchymal transition during interaction. Cell Commun. Signal..

[36] Shi W., Cassmann T.J., Bhagwate A.V., Hitosugi T., Ip W.E. (2024). Lactic acid induces transcriptional repression of macrophage inflammatory response via histone acetylation. Cell Rep..

[37] Zhang D., Tang Z., Huang H., Zhou G., Cui C., Weng Y., Liu W., Kim S., Lee S., Perez-Neut M. (2019). Metabolic regulation of gene expression by histone lactylation. Nature.

[38] Zhao J., Chen L., Shu B., Tang J., Zhang L., Xie J., Qi S., Xu Y. (2014). Granulocyte/macrophage colony-stimulating factor influences angiogenesis by regulating the coordinated expression of VEGF and the Ang/Tie system. PLoS ONE.

[39] Song Y., Tang C., Yin C. (2018). Combination antitumor immunotherapy with VEGF and PIGF siRNA via systemic delivery of multi-functionalized nanoparticles to tumor-associated macrophages and breast cancer cells. Biomaterials.

[40] Guo X., Zhang H., He C., Qin K., Lai Q., Fang Y., Chen Q., Li W., Wang Y., Wang X. (2024). RUNX1 promotes angiogenesis in colorectal cancer by regulating the crosstalk between tumor cells and tumor associated macrophages. Biomark. Res..

[41] Harney A.S., Karagiannis G.S., Pignatelli J., Smith B.D., Kadioglu E., Wise S.C., Hood M.M., Kaufman M.D., Leary C.B., Lu W.-P. (2017). The selective Tie2 inhibitor rebastinib blocks recruitment and function of Tie2Hi macrophages in breast cancer and pancreatic neuroendocrine tumors. Mol. Cancer Ther..

[42] Duran C.L., Borriello L., Karagiannis G.S., Entenberg D., Oktay M.H., Condeelis J.S. (2021). Targeting Tie2 in the tumor microenvironment: From angiogenesis to dissemination. Cancers.

[43] Lu Y., Han G., Zhang Y., Zhang L., Li Z., Wang Q., Chen Z., Wang X., Wu J. (2023). M2 macrophage-secreted exosomes promote metastasis and increase vascular permeability in hepatocellular carcinoma. Cell Commun. Signal..

[44] Gadde M., Mehrabi-Dehdezi M., Debeb B.G., Woodward W.A., Rylander M.N. (2023). Influence of Macrophages on Vascular Invasion of Inflammatory Breast Cancer Emboli Measured Using an In Vitro Microfluidic Multi-Cellular Platform. Cancers.

[45] Miller T.W., Hennessy B.T., González-Angulo A.M., Fox E.M., Mills G.B., Chen H., Higham C., García-Echeverría C., Shyr Y., Arteaga C.L. (2010). Hyperactivation of phosphatidylinositol-3 kinase promotes escape from hormone dependence in estrogen receptor–positive human breast cancer. J. Clin. Investig..

[46] Takebe N., Harris P.J., Warren R.Q., Ivy S.P. (2010). Targeting cancer stem cells by inhibiting Wnt, Notch, and Hedgehog pathways. Nat. Rev. Clin. Oncol..

[47] Xiao M., He J., Yin L., Chen X., Zu X., Shen Y. (2021). Tumor-associated macrophages: Critical players in drug resistance of breast cancer. Front. Immunol..

[48] Su P., Jiang L., Zhang Y., Yu T., Kang W., Liu Y., Yu J. (2022). Crosstalk between tumor-associated macrophages and tumor cells promotes chemoresistance via CXCL5/PI3K/AKT/mTOR pathway in gastric cancer. Cancer Cell Int..

[49] Yang C., He L., He P., Liu Y., Wang W., He Y., Du Y. (2015). Increased drug resistance in breast cancer by tumor-associated macrophages through IL-10/STAT3/bcl-2 signaling pathway. Med. Oncol..

[50] Dean M. (2009). ABC transporters, drug resistance, and cancer stem cells. J. Mammary Gland. Biol. Neoplasia.

[51] Hesse M., Modolell M., La Flamme A.C., Schito M., Fuentes J.M., Cheever A.W., Pearce E.J., Wynn T.A. (2001). Differential regulation of nitric oxide synthase-2 and arginase-1 by type 1/type 2 cytokines in vivo: Granulomatous pathology is shaped by the pattern of L-arginine metabolism. J. Immunol..

[52] Fu X.-T., Song K., Zhou J., Shi Y.-H., Liu W.-R., Shi G.-M., Gao Q., Wang X.-Y., Ding Z.-B., Fan J. (2019). Tumor-associated macrophages modulate resistance to oxaliplatin via inducing autophagy in hepatocellular carcinoma. Cancer Cell Int..

[53] Yu S., Li Q., Yu Y., Cui Y., Li W., Liu T., Liu F. (2020). Activated HIF1α of tumor cells promotes chemoresistance development via recruiting GDF15-producing tumor-associated macrophages in gastric cancer. Cancer Immunol. Immunother..

[54] Liu Z., Kuang W., Zhou Q., Zhang Y. (2018). TGF-β1 secreted by M2 phenotype macrophages enhances the stemness and migration of glioma cells via the SMAD2/3 signalling pathway. Int. J. Mol. Med..

[55] Jinushi M., Chiba S., Yoshiyama H., Masutomi K., Kinoshita I., Dosaka-Akita H., Yagita H., Takaoka A., Tahara H. (2011). Tumor-associated macrophages regulate tumorigenicity and anticancer drug responses of cancer stem/initiating cells. Proc. Natl. Acad. Sci. USA.

[56] Li H., Luo F., Jiang X., Zhang W., Xiang T., Pan Q., Cai L., Zhao J., Weng D., Li Y. (2022). CircITGB6 promotes ovarian cancer cisplatin resistance by resetting tumor-associated macrophage polarization toward the M2 phenotype. J. Immunother. Cancer.

[57] Ma J., Shayiti F., Ma J., Wei M., Hua T., Zhang R., Su J., Chen P. (2021). Tumor-associated macrophage-derived CCL5 promotes chemotherapy resistance and metastasis in prostatic cancer. Cell Biol. Int..

[58] Baghdadi M., Wada H., Nakanishi S., Abe H., Han N., Putra W.E., Endo D., Watari H., Sakuragi N., Hida Y. (2016). Chemotherapy-induced IL34 enhances immunosuppression by tumor-associated macrophages and mediates survival of chemoresistant lung cancer cells. Cancer Res..

[59] Li D., Ji H., Niu X., Yin L., Wang Y., Gu Y., Wang J., Zhou X., Zhang H., Zhang Q. (2019). Tumor-associated macrophages secrete CC-chemokine ligand 2 and induce tamoxifen resistance by activating PI3K/Akt/mTOR in breast cancer. Cancer Sci..

[60] Zheng P., Chen L., Yuan X., Luo Q., Liu Y., Xie G., Ma Y., Shen L. (2017). Exosomal transfer of tumor-associated macrophage-derived miR-21 confers cisplatin resistance in gastric cancer cells. J. Exp. Clin. Cancer Res..

[61] Curiel T.J., Coukos G., Zou L., Alvarez X., Cheng P., Mottram P., Evdemon-Hogan M., Conejo-Garcia J.R., Zhang L., Burow M. (2004). Specific recruitment of regulatory T cells in ovarian carcinoma fosters immune privilege and predicts reduced survival. Nat. Med..

[62] Bian Z., Shi L., Kidder K., Zen K., Garnett-Benson C., Liu Y. (2021). Intratumoral SIRPα-deficient macrophages activate tumor antigen-specific cytotoxic T cells under radiotherapy. Nat. Commun..

[63] Kamat K., Krishnan V., Dorigo O. (2022). Macrophage-derived CCL23 upregulates expression of T-cell exhaustion markers in ovarian cancer. Br. J. Cancer.

[64] Peranzoni E., Lemoine J., Vimeux L., Feuillet V., Barrin S., Kantari-Mimoun C., Bercovici N., Guérin M., Biton J., Ouakrim H. (2018). Macrophages impede CD8 T cells from reaching tumor cells and limit the efficacy of anti–PD-1 treatment. Proc. Natl. Acad. Sci. USA.

[65] Pu J., Xu Z., Nian J., Fang Q., Yang M., Huang Y., Li W., Bin Ge B., Wang J., Wei H. (2021). M2 macrophage-derived extracellular vesicles facilitate CD8^+^ T cell exhaustion in hepatocellular carcinoma via the miR-21-5p/YOD1/YAP/β-catenin pathway. Cell Death Discov..

[66] Miller B.C., Sen D.R., Al Abosy R., Bi K., Virkud Y.V., LaFleur M.W., Yates K.B., Lako A., Felt K., Naik G.S. (2019). Subsets of exhausted CD8+ T cells differentially mediate tumor control and respond to checkpoint blockade. Nat. Immunol..

[67] Xu Y., Zeng H., Jin K., Liu Z., Zhu Y., Xu L., Wang Z., Chang Y., Xu J. (2022). Immunosuppressive tumor-associated macrophages expressing interlukin-10 conferred poor prognosis and therapeutic vulnerability in patients with muscle-invasive bladder cancer. J. Immunother. Cancer.

[68] Kersten K., Hu K.H., Combes A.J., Samad B., Harwin T., Ray A., Rao A.A., Cai E., Marchuk K., Artichoker J. (2022). Spatiotemporal co-dependency between macrophages and exhausted CD8+ T cells in cancer. Cancer Cell.

[69] Yan D., Wang H.-W., Bowman R.L., Joyce J.A. (2016). STAT3 and STAT6 signaling pathways synergize to promote cathepsin secretion from macrophages via IRE1α activation. Cell Rep..

[70] Deryugina E.I., Zajac E., Juncker-Jensen A., Kupriyanova T.A., Welter L., Quigley J.P. (2014). Tissue-infiltrating neutrophils constitute the major in vivo source of angiogenesis-inducing MMP-9 in the tumor microenvironment. Neoplasia.

[71] Petty A.J., Owen D.H., Yang Y., Huang X. (2021). Targeting tumor-associated macrophages in cancer immunotherapy. Cancers.

[72] Häuselmann I., Roblek M., Protsyuk D., Huck V., Knopfova L., Grässle S., Bauer A.T., Schneider S.W., Borsig L. (2016). Monocyte Induction of E-selectin–mediated endothelial activation releases VE-cadherin junctions to promote tumor cell extravasation in the metastasis cascade. Cancer Res.

[73] Glass E.B., Hoover A.A., Bullock K.K., Madden M.Z., Reinfeld B.I., Harris W., Parker D., Hufnagel D.H., Crispens M.A., Khabele D. (2022). Stimulating TAM-mediated anti-tumor immunity with mannose-decorated nanoparticles in ovarian cancer. BMC Cancer.

[74] Tu M.M., Abdel-Hafiz H.A., Jones R.T., Jean A., Hoff K.J., Duex J.E., Chauca-Diaz A., Costello J.C., Dancik G.M., Tamburini B.A.J. (2020). Inhibition of the CCL2 receptor, CCR2, enhances tumor response to immune checkpoint therapy. Commun. Biol..

[75] Cai J., Xia L., Li J., Ni S., Song H., Wu X. (2019). Tumor-associated macrophages derived TGF-β—induced epithelial to mesenchymal transition in colorectal cancer cells through Smad2,3-4/Snail signaling pathway. Cancer Res. Treat..

[76] Fu X.-T., Dai Z., Song K., Zhang Z.-J., Zhou Z.-J., Zhou S.-L., Zhao Y.-M., Xiao Y.-S., Sun Q.-M., Ding Z.-B. (2015). Macrophage-secreted IL-8 induces epithelial-mesenchymal transition in hepatocellular carcinoma cells by activating the JAK2/STAT3/Snail pathway. Int. J. Oncol..

[77] Gomez-Roca C., Cassier P., Zamarin D., Machiels J.-P., Gracia J.L.P., Hodi F.S., Taus A., Garcia M.M., Boni V., Eder J.P. (2022). Anti-CSF-1R emactuzumab in combination with anti-PD-L1 atezolizumab in advanced solid tumor patients naïve or experienced for immune checkpoint blockade. J. Immunother. Cancer.

[78] Cassier P.A., Italiano A., Gomez-Roca C., Le Tourneau C., Toulmonde M., D’Angelo S.P., Weber K., Loirat D., Jacob W., Jegg A.-M. (2020). Long-term clinical activity, safety and patient-reported quality of life for emactuzumab-treated patients with diffuse-type tenosynovial giant-cell tumour. Eur. J. Cancer.

[79] Gomez-Roca C.A., Italiano A., Le Tourneau C., Cassier P.A., Toulmonde M., D’Angelo S.P., Campone M., Weber K.L., Loirat D., Cannarile M.A. (2019). Phase I study of emactuzumab single agent or in combination with paclitaxel in patients with advanced/metastatic solid tumors reveals depletion of immunosuppressive M2-like macrophages. Ann. Oncol..

[80] Djureinovic D., Weiss S.A., Krykbaeva I., Qu R., Vathiotis I., Moutafi M., Zhang L., Perdigoto A.L., Wei W., Anderson G. (2023). A bedside to bench study of anti-PD-1, anti-CD40, and anti-CSF1R indicates that more is not necessarily better. Mol. Cancer.

[81] Kuemmel S., Campone M., Loirat D., Lopez R.L., Beck J.T., De Laurentiis M., Im S.-A., Kim S.-B., Kwong A., Steger G.G. (2022). A randomized phase II study of anti-CSF1 monoclonal antibody lacnotuzumab (MCS110) combined with gemcitabine and carboplatin in advanced triple-negative breast cancer. Clin. Cancer Res..

[82] Nywening T.M., Wang-Gillam A., Sanford D.E., Belt B.A., Panni R.Z., Cusworth B.M., Toriola A.T., Nieman R.K., Worley L.A., Yano M. (2016). Phase 1b study targeting tumour associated macrophages with CCR2 inhibition plus FOLFIRINOX in locally advanced and borderline resectable pancreatic cancer. Lancet Oncol..

[83] Abdelgalil A.A., Alkahtani H.M., Al-Jenoobi F.I. (2019). Sorafenib. Profiles of Drug Substances, Excipients, and Related Methodology.

[84] Yamada T., Fujiwara N., Kubota N., Matsushita Y., Nakatsuka T., Kurosaki S., Minami T., Tateishi R., Ichida A., Arita J. (2023). Lenvatinib recruits cytotoxic GZMK+CD8 T cells in hepatocellular carcinoma. Hepatol. Commun..

[85] Kudo M., Finn R.S., Qin S., Han K.-H., Ikeda K., Piscaglia F., Baron A., Park J.-W., Han G., Jassem J. (2018). Lenvatinib versus sorafenib in first-line treatment of patients with unresectable hepatocellular carcinoma: A randomised phase 3 non-inferiority trial. Lancet.

[86] Reid T., Oronsky B., Caroen S., Quinn M., Williams J., Cabrales P., Abrouk N. (2023). Phase 1 pilot study of RRx-001 + nivolumab in patients with advanced metastatic cancer (PRIMETIME). Front. Immunol..

[87] Byrne K.T., Betts C.B., Mick R., Sivagnanam S., Bajor D.L., Laheru D.A., Chiorean E.G., O’Hara M.H., Liudahl S.M., Newcomb C.W. (2021). Neoadjuvant selicrelumab, an agonist CD40 antibody, induces changes in the tumor microenvironment in patients with resectable pancreatic cancer. Clin. Cancer Res..

[88] Luke J.J., Barlesi F., Chung K., Tolcher A.W., Kelly K., Hollebecque A., Le Tourneau C., Subbiah V., Tsai F., Kao S. (2021). Phase I study of ABBV-428, a mesothelin-CD40 bispecific, in patients with advanced solid tumors. J. Immunother. Cancer.

[89] Li J., Qin S., Xu J., Xiong J., Wu C., Bai Y., Liu W., Tong J., Liu Y., Xu R. (2016). Randomized, double-blind, placebo-controlled phase III trial of apatinib in patients with chemotherapy-refractory advanced or metastatic adenocarcinoma of the stomach or gastroesophageal junction. J. Clin. Oncol..

[90] Zhao S., Ren S., Jiang T., Zhu B., Li X., Zhao C., Jia Y., Shi J., Zhang L., Liu X. (2019). Low-dose apatinib optimizes tumor microenvironment and potentiates antitumor effect of PD-1/PD-L1 blockade in lung cancer. Cancer Immunol. Res..

[91] Rajan A., Sater H.A., Rahma O., Agajanian R., Lassoued W., Marté J.L., Tsai Y.-T., Donahue R.N., Lamping E., Bailey S. (2024). Efficacy, safety, and biomarker analyses of bintrafusp alfa, a bifunctional fusion protein targeting TGF-β and PD-L1, in patients with advanced non-small cell lung cancer. J. Immunother. Cancer.

[92] Chen Y., Zhu X., Liu H., Wang C., Chen Y., Wang H., Fang Y., Wu X., Xu Y., Li C. (2023). The application of HER2 and CD47 CAR-macrophage in ovarian cancer. J. Transl. Med..

[93] Llovet J.M., Montal R., Sia D., Finn R.S. (2018). Molecular therapies and precision medicine for hepatocellular carcinoma. Nat. Rev. Clin. Oncol..

[94] Wilhelm S., Carter C., Lynch M., Lowinger T., Dumas J., Smith R.A., Schwartz B., Simantov R., Kelley S. (2006). Discovery and development of sorafenib: A multikinase inhibitor for treating cancer. Nat. Rev. Drug Discov..

[95] Huang L., Xu R., Li W., Lv L., Lin C., Yang X., Yao Y., Saw P.E., Xu X. (2023). Repolarization of macrophages to improve sorafenib sensitivity for combination cancer therapy. Acta Biomater..

[96] Tang W., Chen Z., Zhang W., Cheng Y., Zhang B., Wu F., Wang Q., Wang S., Rong D., Reiter F.P. (2020). The mechanisms of sorafenib resistance in hepatocellular carcinoma: Theoretical basis and therapeutic aspects. Signal Transduct. Target. Ther..

[97] Parayath N.N., Parikh A., Amiji M.M. (2018). Repolarization of tumor-associated macrophages in a genetically engineered nonsmall cell lung cancer model by intraperitoneal administration of hyaluronic acid-based nanoparticles encapsulating microRNA-125b. Nano Lett..

[98] Parayath N.N., Gandham S.K., Leslie F., Amiji M.M. (2019). Improved anti-tumor efficacy of paclitaxel in combination with MicroRNA-125b-based tumor-associated macrophage repolarization in epithelial ovarian cancer. Cancer Lett..

[99] Zhang H., Mao Y., Nie Z., Li Q., Wang M., Cai C., Hao W., Shen X., Gu N., Shen W. (2024). Iron Oxide Nanoparticles Engineered Macrophage-Derived Exosomes for Targeted Pathological Angiogenesis Therapy. ACS Nano.

[100] Negrea G., Rauca V.-F., Meszaros M.S., Patras L., Luput L., Licarete E., Toma V.-A., Porfire A., Muntean D., Sesarman A. (2022). Active tumor-targeting nano-formulations containing simvastatin and doxorubicin inhibit melanoma growth and angiogenesis. Front. Pharmacol..

[101] Tian L., Yi X., Dong Z., Xu J., Liang C., Chao Y., Wang Y., Yang K., Liu Z. (2018). Calcium bisphosphonate nanoparticles with chelator-free radiolabeling to deplete tumor-associated macrophages for enhanced cancer radioisotope therapy. ACS Nano.

[102] Ramesh A., Kumar S., Nandi D., Kulkarni A. (2019). CSF1R-and SHP2-inhibitor-loaded nanoparticles enhance cytotoxic activity and phagocytosis in tumor-associated macrophages. Adv. Mater..

[103] Sun Z., Li R., Sun J., Peng Y., Xiao L., Zhang X., Xu Y., Wang M. (2017). Matrix metalloproteinase cleavable nanoparticles for tumor microenvironment and tumor cell dual-targeting drug delivery. ACS Appl. Mater. Interfaces.

[104] Zhang Y., Feng Z., Liu J., Li H., Su Q., Zhang J., Huang P., Wang W., Liu J. (2022). Polarization of tumor-associated macrophages by TLR7/8 conjugated radiosensitive peptide hydrogel for overcoming tumor radioresistance. Bioact. Mater..

[105] Song M., Liu T., Shi C., Zhang X., Chen X. (2015). Bioconjugated manganese dioxide nanoparticles enhance chemotherapy response by priming tumor-associated macrophages toward M1-like phenotype and attenuating tumor hypoxia. ACS Nano.

[106] Zhang X., Tian W., Cai X., Wang X., Dang W., Tang H., Cao H., Wang L., Chen T. (2013). Hydrazinocurcumin encapsuled nanoparticles “re-educate” tumor-associated macrophages and exhibit anti-tumor effects on breast cancer following STAT3 suppression. PLoS ONE.

[107] Han S., Wang W., Wang S., Yang T., Zhang G., Wang D., Ju R., Lu Y., Wang H., Wang L. (2021). Tumor microenvironment remodeling and tumor therapy based on M2-like tumor associated macrophage-targeting nano-complexes. Theranostics.

[108] Chen C., Song M., Du Y., Yu Y., Li C., Han Y., Yan F., Shi Z., Feng S. (2021). Tumor-associated-macrophage-membrane-coated nanoparticles for improved photodynamic immunotherapy. Nano Lett..

[109] Qian Y., Qiao S., Dai Y., Xu G., Dai B., Lu L., Yu X., Luo Q., Zhang Z. (2017). Molecular-targeted immunotherapeutic strategy for melanoma via dual-targeting nanoparticles delivering small interfering RNA to tumor-associated macrophages. ACS Nano.

[110] Kaps L., Leber N., Klefenz A., Choteschovsky N., Zentel R., Nuhn L., Schuppan D. (2020). In vivo siRNA delivery to immunosuppressive liver macrophages by α-mannosyl-functionalized cationic nanohydrogel particles. Cells.

[111] Liu L., He H., Liang R., Yi H., Meng X., Chen Z., Pan H., Ma Y., Cai L. (2018). ROS-inducing micelles sensitize tumor-associated macrophages to TLR3 stimulation for potent immunotherapy. Biomacromolecules.

[112] Wang L., Hu Y.-Y., Zhao J.-L., Huang F., Liang S.-Q., Dong L., Chen Y., Yu H.-C., Bai J., Yang J.-M. (2020). Targeted delivery of miR-99b reprograms tumor-associated macrophage phenotype leading to tumor regression. J. Immunother. Cancer.

[113] D’Urso A., Oltolina F., Borsotti C., Prat M., Colangelo D., Follenzi A. (2023). Macrophage Reprogramming via the Modulation of Unfolded Protein Response with siRNA-Loaded Magnetic Nanoparticles in a TAM-like Experimental Model. Pharmaceutics.

[114] Qian H., Zhou T., Fu Y., Guo M., Yang W., Zhang D., Fang W., Yao M., Shi H., Chai C. (2022). Self-assembled tetrahedral framework nucleic acid mediates tumor-associated macrophage reprogramming and restores antitumor immunity. Mol. Ther. Nucleic Acids.

[115] Liu H., Huang L., Mao M., Ding J., Wu G., Fan W., Yang T., Zhang M., Huang Y., Xie H. (2020). Viral Protein-Pseudotyped and siRNA-Electroporated Extracellular Vesicles for Cancer Immunotherapy. Adv. Funct. Mater..

[116] Egorova V.S., Kolesova E.P., Lopus M., Yan N., Parodi A., Zamyatnin A.A. (2023). Smart Delivery Systems Responsive to Cathepsin B Activity for Cancer Treatment. Pharmaceutics.

[117] Liao D., Liu Z., Wrasidlo W., Chen T., Luo Y., Xiang R., Reisfeld R.A. (2011). Synthetic enzyme inhibitor: A novel targeting ligand for nanotherapeutic drug delivery inhibiting tumor growth without systemic toxicity. Nanomed. Nanotechnol. Biol. Med..

